# The use of granulocyte colony-stimulating factors in the management of breast cancer patients

**DOI:** 10.3389/fonc.2025.1613199

**Published:** 2025-09-09

**Authors:** Anna Bałata, Ewa Szombara, Zbigniew I. Nowecki, Katarzyna Pogoda

**Affiliations:** Department of Breast Cancer and Reconstructive Surgery, Maria Sklodowska-Curie National Research Institute of Oncology, Warsaw, Poland

**Keywords:** breast cancer, neutropenia, febrile neutropenia, granulocyte colony-stimulating factors, filgrastim, pegfilgrastim, anticancer therapy toxicity

## Abstract

The dynamic development of oncology poses new challenges to health care practitioners (HCPs), related to the introduction of modern targeted therapies, immunotherapies, or new chemotherapy regimens. The previous treatment algorithm for neutropenia and febrile neutropenia have significantly reduced the frequency of these complications in long-term therapies. Currently, breast cancer (BC) treatment is also based on the use of modern therapies, showing different toxicity profile. This article discusses the role of granulocyte colony-stimulating factors (G-CSFs) in well-known and new therapies used in the treatment of breast cancer patients. The factors influencing the development of hematological complications such as neutropenia and febrile neutropenia were presented. An important aspect of the assessment of patients at increased risk of therapy-induced toxicity was discussed with highlighting the fact that the treatment regimen is not the only factor influencing the development of adverse events (AEs). The aim of the study is to consolidate recommendations for the primary and secondary prevention of neutropenia and febrile neutropenia and related improvement of treatment outcomes in patients with BC.

## Introduction

Breast cancer is the most frequently diagnosed malignant tumor in women. The current decrease in mortality results from early diagnosis thanks to modern screening techniques and advances in local and systemic treatment (1). However, targeted therapies, immunotherapy, chemotherapy regimens with a shortened interval between cycles are associated with a different toxicity profile. Therefore, there is a need for developing new management algorithms in everyday clinical practice. Appropriate management of adverse effects during the treatment of early and advanced BC is an important element of anticancer therapy and directly improves prognosis.

## Neutropenia and febrile neutropenia

Neutropenia, defined as a decrease in the absolute number of peripheral blood neurophils <1500 G/L, is a frequent adverse effect of anticancer treatment (2). According to the European Society for Medical Oncology (ESMO), febrile neutropenia (FN), is characterized by an absolute decrease in neurophils count below 500 G/L or a predicted decrease in their number below 500 G/L with an associated increase in body temperature above 38.3°C or above 38°C in two consecutive measurements within 2 hours (2).

Ten years ago, FN occurred in up to 17% of BC patients receiving systemic therapies (3). The risk of this complication depends not only on the treatment regimen used, but also other factors. Elderly patients, especially with comorbidities, advanced disease stages and FN in medical history are at increased risk of developing this complication (4).

Appropriate assessment of patient is of significant importance in decision making process regarding G-CSF prophylaxis. The risk of FN-related complications in BC patients can be estimated using the MASCC (Multinational Association of Supportive Care in Cancer) risk index. A score of ≥ 21 is considered as low risk, with the frequency of serious complications of 6% (probability of death - 1%), and if number of points is below 21, the risk of serious complications is as high as 39% (risk of death - 14%) (5, 6).

It has been shown that patients with bacteremia have a higher risk of death related to FN. In patients with Gram-negative and Gram-positive bacteria in blood cultures, mortality increases to 18% and 5%, respectively (7). The etiology of bacteremia has additional prognostic value, especially in patients at high risk of complications (6).

## Granulocyte colony-stimulating factors

### Filgrastim

The first available G-CSF was a short-acting recombinant methionyl human granulocyte colony-stimulating factor filgrastim. Clinical trials evaluating its activity and safety in patients undergoing cytotoxic therapy began in 1988 (8). The dose of filgrastim is 0.5 million units (5 μg)/kg of body weight (b.w.)/day. The first dose should not be administered earlier than 24 hours after chemotherapy completion. A transient increase in neurocyte counts may usually be observed 1–2 days after therapy initiation. However, to achieve a sustained response, filgrastim should not be discontinued before the expected nadir and the neutrophil count recovery. Filgrastim should be administered approximately 10–14 days during chemotherapy with 3-week or longer cycles (9).

According to the retrospective study, the use of filgrastim reduces the risk of G3/G4 neutropenia or shortens its duration in patients undergoing chemotherapy, compared with placebo or no intervention. It significantly reduces the risk of FN and the number of FN-related hospitalizations, the use of antibiotics, and the number of deaths (10).

G-CSF has a direct impact on the effectiveness of anticancer treatment because it affects the proper course of systemic treatment over time without reducing the doses. However, none of the studies assessing the efficacy of filgrastim found its effect on prolonging overall survival (OS) or disease-free survival (DFS). The characteristic adverse effects of filgrastim were generalized bone pain and flu-like symptoms (11).

### Pegfilgrastim

Given the short half-life of filgrastim and the need for daily dosing, an attempt was made to modify this molecule, resulting in a long-acting, newer-generation drug. Pegfilgrastim is a covalent conjugate of recombinant human G-CSF with one molecule of polyethylene glycol, with reduced plasma clearance and extended half-life to 80 hours. This allows for reduction of the frequency of G-CSF administration to a single dose during chemotherapy cycle while maintaining previous efficacy (6 mg per chemotherapy cycle, administered at least 24 hours after treatment cessation) (6, 12). The drug was registered by the US Food and Drugs Administration (FDA) and the European Medicines Agency (EMA) in 2002 based on the results of two phase III studies. The first study assessed the efficacy and safety of pegfilgrastim compared with filgrastim in BC patients undergoing neoadjuvant chemotherapy according to AT regimen. A single dose of pegfilgrastim was as effective as 10 injections of filgrastim in reducing the risk and duration of grade 3 and 4 neutropenia. The frequency of FN during 4 cycles of chemotherapy was lower in pegfilgrastim group (9% vs. 18%) (13).

The second study compared the efficacy and safety of pegfilgrastim and filgrastim in 157 BC patients receiving combined chemotherapy with docetaxel and doxorubicin. The incidence of G4 neutropenia was similar between the two groups. FN occurred in 13% of patients receiving pegfilgrastim compared with 20% of patients receiving filgrastim (14).

In majority of the studies the use of pegfilgrastim resulted in a lower risk of neutropenia and FN. According to meta-analysis of 5 studies, including 617 patients, also with BC, a single dose of pegfilgrastim was significantly more effective in minimizing the risk of G3/G4 neutropenia and FN compared to 14 days of filgrastim therapy (15). Similar results were obtained in Cooper et al. meta-analysis of 20 studies, including over 4,000 cancers patients undergoing systemic therapies (16, 61).

These findings are consistent with those of Li et al. (2020), who in a systematic review and meta-analysis demonstrated that PEGylated G-CSF significantly reduces the incidence of febrile neutropenia and is at least as effective, if not superior, to filgrastim in breast cancer patients receiving chemotherapy (17).

In Cornes et al. meta-analysis of 11 randomized clinical trials and 2 non-randomized studies, no significant advantage of long-acting over short-acting preparations was observed in reducing the incidence of FN (18). One of the hypotheses explaining the contradictory results is the lower efficacy of short-acting preparations resulting from a lower total dose compared to a single injection of pegfilgrastim. It is estimated that 6 mg of pegfilgrastim is equivalent to 11 filgrastim administrations (6, 18). in addition, a single injection of pegfilgrastim does not significantly reduce QoL, compared to therapy lasting at least 10 days in each chemotherapy cycle. It is important to note that, compared to filgrastim, pegfilgrastim is not compatible with a weekly chemotherapy regimen.

## Primary and secondary prophylaxis

**Figure 1:**
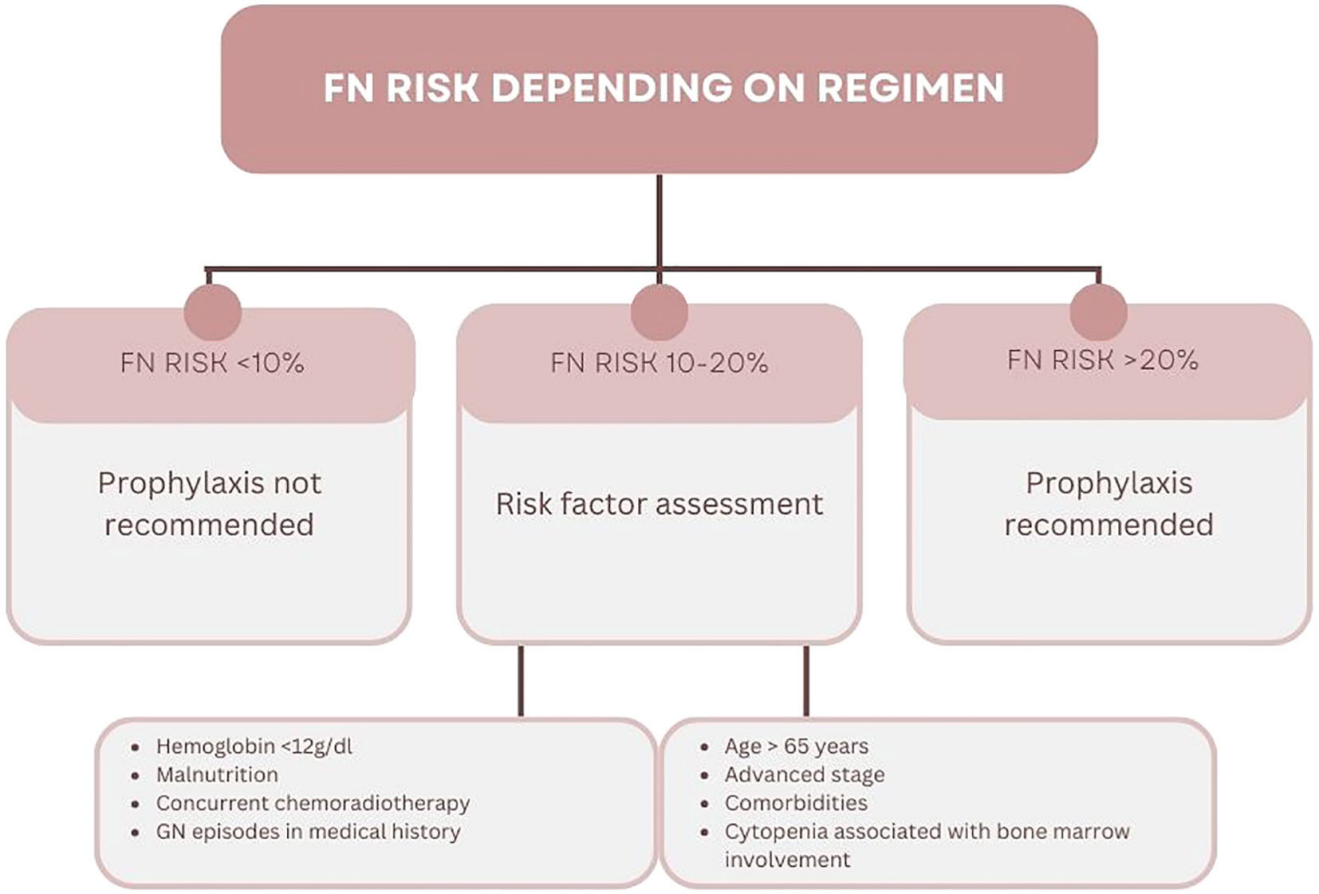
Stratification of febrile neutropenia risk groups (2, 6).

G-CSF therapy may be implemented as primary or secondary prophylaxis. The latter is introducing in patients with G3/G4 neutropenia, FN or FN-related infection during cytotoxic therapy. This prevents the occurrence of neutropenia or FN or shortens their duration. During primary prophylaxis, the patient receives G-CSF after the first course of chemotherapy due to the primary high risk of FN. It is recommended to implement primary prophylaxis in patients receiving treatment according to regimens that are associated with FN risk greater than 20%. An intermediate risk of FN, i.e., 10-20%, does not require the absolute use of G-CSF prophylaxis (**Figure 1**) (2, 6).

Important factors that should be taken into account include patient’s age and performance status (PS) according to the Eastern Cooperative Oncology Group (ECOG) score. A significant increase in FN risk is also associated with comorbidities, such as chronic renal failure, liver failure or heart disease. The frequency of hematological complications, including FN, is significantly higher in patients receiving intensive treatment due to advanced cancer as compared to patients with early stages. However, each clinical situation should be assessed individually (2, 6).

The utility of primary G-CSF prophylaxis has also been confirmed in a recent meta-analysis by Nozawa et al. (2024), who concluded that such prophylaxis significantly reduces the risk of febrile neutropenia without compromising the safety profile in patients with invasive breast cancer (19).

## Use of GCS-F during chemotherapy

The current systemic treatment of BC patients should be individualized, based on many factors, including clinical stage, biological subtype, *BRCA* mutation status, programmed death ligand 1 (PD-L1) expression level, *PIK3Ca* mutation status and patient’s general condition. However, chemotherapy is still one of the basic treatment modalities (16, 20).

In patients with triple negative and human epidermal growth factor receptor 2 [HER2] -positive early breast cancer (EBC), not achieving pathological complete response (pCR), further systemic treatment is implemented in addition to neoadjuvant therapy (18). Similarly, HR+/HER2− patients at high risk of recurrence—whether treated with neoadjuvant endocrine therapy or primarily eligible for surgery—also require adjuvant chemotherapy. Unfortunately, this prolongs the therapeutic process and increases the risk of additional toxicities. Therefore, minimizing the risk of AEs (including FN) and the resulting delays in treatment cycles and dose reductions has a significant impact on treatment of patients with EBC (21).

### Chemotherapy in early breast cancer

The therapy based on anthracyclines and taxane, being a “gold standard” perioperative treatment of BC patients, is associated with 10-20% risk of FN (**Table 1**). Primary prophylaxis with G-CSF is therefore not recommended for all patients receiving this regimen. Currently, the standard anthracycline treatment regimen, i.e., every 3 weeks, is reserved for patients with low disease dynamics and additional disease burden. Remaining patients require intensive chemotherapy regimen (dose dense, dd), which is associated with >20% risk of FN. It was shown in the randomized clinical trial, that anthracyclines administered every 14 days (Q2W) prolonged DFS and OS compared to the conventional rhythm of therapy. The administration of G-CSF as primary prophylaxis (3–10 days between treatment cycles) was associated with the requirement to maintain 14-day therapy cycles. Dose-dense regimens were associated with a lower frequency of FN and G3/G4 neutropenia, compared to the conventional rhythm of treatment without G-CSF prophylaxis (6% vs 33%, respectively). Additionally, there were more treatment delays during the 3-week treatment regimen compared to the 2-week regimen (38% vs 15%, respectively) (22).

**Table 1:** The risk of febrile neutropenia in BC patients depending on the systemic treatment regimen (2, 16).

FN risk	Treatment regimen
FN RISK >20%	• TCH-P (docetaxel, carboplatin, trastuzumab, pertuzumab) • TCH (docetaxel, carboplatin, trastuzumab), • TC (docetaxel, cyclophosphamide), • ddAC → T (dose dense doxorubicin + cyclophosphamide - paclitaxel) (only AC part)
FN RISK 10-20%	• AC→T (doxorubicin + cyclophosphamide ->paclitaxel) (only AC q3w part) • PTH (pertuzumab, trastuzumab, docetaxel) • Docetaxel - every 21 days • Sacituzumab govitecan
FN RISK <10%	• CMF (cyclophosphamide, methotrexate, fluorouracil) • Paclitaxel weekly, capecitabine, vinorelbine, gemcitabine, carboplatin • Trastuzumab, pertuzumab • Trastuzumab emtansine • Trastuzumab deruxtecan • Lapatinib, tucatinib • Palbociclib, ribociclib, abemaciclib • Pembrolizumab, atezolizumab • Olaparib, talazoparib • Alpelisib

For some patients the dose-dense treatment regimen may be too intense, taking into account patient’s age or comorbidities. It can therefore be stated that such patients are also at higher risk of hematological toxicity and are also candidates for primary FN prophylaxis.

The importance of G-CSF support in dose-dense chemotherapy was also emphasized in the meta-analysis by Yokoe et al. (2025), which confirmed the efficacy and safety of pegfilgrastim prophylaxis in patients with early-stage breast cancer undergoing dose-dense regimens, supporting its routine use in this setting (23).

Another regimen of chemotherapy is combination of docetaxel with cyclophosphamide (TC). In a multicenter, randomized phase II study, the frequency of FN during 6 treatment cycles was significantly lower in the group receiving pegfilgrastim compared to the group without primary prophylaxis (1.2% vs. 68.8%, respectively) (24). It shows that TC regimen is also associated with the highest risk of FN and requires primary prophylaxis with G-CSF is (24). One of the currently recommended treatment regimens for patients with HER2-positive EBC is a combination of docetaxel, carboplatin, and trastuzumab with or without pertuzumab (TCH+/-P) (25).

Numerous studies have assessed the risk of FN during treatment with TCH+/-P regimen. A meta-analysis of 17 studies found that in patients without primary G-CSF prophylaxis, the incidence of FN was 27.6% (95% CI 18.6 to 37.1) compared to 5,0% (95% CI 2.6 to 8.0) in patients receiving G-CSF after each therapy cycle. The TCH+/-P regimen is therefore associated with >20% risk of FN (25–27). According to NCCN and ESMO recommendations, primary prophylaxis is indicated in patients receiving TCH+/-P regimen (2, 6).

Weekly chemotherapy cycles, used in perioperative treatment and in the treatment of advanced BC, do not significantly increase the risk of FN or G3/G4 neutropenia. NCCN guidelines emphasize that in the case of neoadjuvant treatment based on anthracyclines and paclitaxel, further weekly taxane therapy after completion of 4 cycles of dose-dense AC therapy, does not require the use of primary FN prophylaxis (28).

### Chemotherapy in metastatic breast cancer

Patients with metastatic breast cancer (MBC) receiving carboplatin or paclitaxel in 3-week cycles have significantly higher FN risk (10-20%) compared to 7-day cycles (20, 29). Patients receiving chemotherapy in 21-day cycles should be managed as the group with intermediate FN risk. Therefore, risk factors should be assessed before each subsequent cycle of therapy.

Indications for the use of metronomic chemotherapy in patients with MBC, which involves prolonged, often oral administration of a low-dose cytostatic agent, were presented in the international ABC recommendations in 2017 (30). Due to the better safety profile, this therapy is a good choice especially for elderly patients or with multiple comorbidities.

Metronomic chemotherapy involves oral cyclophosphamide (CTX) in combination with methotrexate (MTX) as well as capecitabine and vinorelbine. The multicenter VICTOR-6 study of metronomic therapies in 584 patients with MBC confirmed a very good safety profile of this treatment. Grade 3/4 hematological toxicities (anemia, thrombocytopenia, leukopenia) were observed in only 5.8% of patients, and no episodes of FN were reported (30). Similar results were obtained in a pooled meta-analysis of 22 studies, which assessed 1360 patients with MBC receiving metronomic therapy (31).

In patients with advanced disease, the current treatment duration is significantly longer than previously. Therefore, the risk of FN increases significantly in this patient. Regular assessment of the general health condition in patients treated palliatively may protect them from severe FN complications.

## The place of granulocyte colony-stimulating factors in new breast cancer therapies

### Triple negative breast cancer

#### Pembrolizumab

Pembrolizumab is a human IgG4 monoclonal antibody that has been used in the treatment of many cancers. The KEYNOTE-522 and KEYNOTE-355 studies have proven its efficacy in triple-negative breast cancer (TNBC), both in perioperative EBC therapies and in MBC treatment (32, 33).

In the KEYNOTE-522 study, evaluating the efficacy of pembrolizumab in combination with paclitaxel and carboplatin followed by a conventional 3-weekly AC regimen, the incidence of grade ≥3 neutropenia was 34.6% compared with 33.2% in patients receiving chemotherapy alone. FN was reported in 14.6% of patients in the experimental arm and 12.1% in the control group. Similar observations were made in the KEYNOTE-355 study, evaluating the efficacy of pembrolizumab in MBC. The incidence of G3 or higher neutropenia was 41.1% in the pembrolizumab group and 38.1% in the group with chemotherapy alone. The incidence of FN was not reported.

Pembrolizumab does not significantly increase the risk of neutropenia and FN. The initiation of primary or secondary prophylaxis using G-CSFs depends only on the chemotherapy component of combination therapy.

#### Atezolizumab

Atezolizumab is a humanized IgG1 monoclonal antibody with a modified Fc region directed against PD-L1. Its efficacy in combination with nab-paclitaxel in the treatment of advanced TNBC was demonstrated in the IMpassion130 study (34). The rate of grade 3/4 neutropenia in the experimental arm was 8.2%, the same as in the control group receiving nab-paclitaxel. There were no FN episodes during the study. Due to the low rate of hematological complications, including neutropenia and FN, the use of G-CSF during atezolizumab treatment is not required.

#### PARP inhibitors (olaparib, talazoparib)

Poly (ADP-ribose) polymerase (PARP) inhibitors have a wide range of indications in patients with HER2-negative BC and confirmed germline *BRCA* mutation (34–36). In clinical trials no significantly higher rates of neutropenia or FN were observed in the experimental arm compared to patients receiving treatment of the investigator’s choice. In the OLYMPIA study, a decrease in the white blood cell count (WBC) of grade 3 or higher was observed in only 3% of patients receiving olaparib as adjuvant therapy,. In the OlympiAD study grade 3/4 neutropenia was more frequent in patients receiving chemotherapy of the investigator’s choice compared to the study group (35, 36).

In the EMBRACA study, incidence of G3/G4 neutropenia in the experimental arm receiving talazoparib was 20.9%, as compared to significantly higher rate in the control group (35.9%) (62). In none of the above studies G-CSF prophylaxis was required. A small percentage of patients with recurrent neutropenia required a reduction in the PARP inhibitor dose, which resulted in normalization of laboratory results.

Olaparib and talazoparib therapy is not associated with a significant risk of G3/G4 neutropenia and FN. Therefore, neither primary nor secondary prophylaxis with G-CSF is required.

#### Sacituzumab govitecan

Sacituzumab govitecan (SG) is a conjugate of the monoclonal antibody sacituzumab and the active metabolite of irinotecan (SN-38 molecule) with cytotoxic activity against topoisomerase I.

Phase III ASCENT study confirmed its efficacy in the treatment of advanced TNBC (37). The drug is also approved for the treatment of unresectable or metastatic luminal HER2-negative BC based on the results of the TROPICS-02 study (38). In both studies, G3/G4 neutropenia was the most common treatment-emergent adverse event in the SG arm. In the ASCENT study, it occurred in 51% of patients, compared with 33% in the chemotherapy arm. FN was reported in 6% of patients in the experimental arm and in 2% of patients in the control group. Similar results were obtained in the TROPICS-02 study (G3/G4 neutropenia - 51% vs 33%; FN - 6% vs 3%, respectively). Due to high rates of neutropenia, it was necessary to reduce the dose of SG or to implement secondary FN prophylaxis. G-CSF was used in 49% of patients in the ASCENT study and in 54% of patients in the TROPICS-02 study. Phase II PRIMED study evaluated the effect of G-CSF on reducing the risk of neutropenia and FN associated with SC therapy. The study included 50 patients who received short-acting G-CSF at a dose of 0.5 mg/kg b.w. on days 3–4 and 10–11 during the first two treatment cycles. Grade 3/4 neutropenia occurred only in 8 patients (16%; p=0.0002), and no case of FN was found in the entire group (39).

Based on presented studies, it is recommended to implement G-CSF prophylaxis in case of recurrent neutropenia during SG therapy. However, it should be noted that in order to continue therapy with SG, it is necessary to obtain at least 1,000 G/L of neutrophils on day 8 of the cycle.

## HER2 positive breast cancer

### Trastuzumab, pertuzumab

The first anti-HER2 drug approved for use in clinical practice was trastuzumab (40, 41). The next step in the development of anti-HER2 therapy was the combination of trastuzumab with another monoclonal antibody, pertuzumab in patients with advanced or MBC. In the CLEOPATRA study, any grade neutropenia, occurred in 53.4% ​​of patients in pertuzumab arm and in 50% of patients in the control group receiving docetaxel in combination with trastuzumab. During further treatment with antibodies alone, neutropenia was observed in 3.3% of patients receiving combined therapy, compared to 5.0% in the control group (42).

FN risk related to trastuzumab and pertuzumab therapy is low, therefore it does not require G-CSF prophylaxis. However, the risk of FN resulting from the chemotherapy used in combination with monoclonal antibodies, should be taken into account.

### Trastuzumab emtansine

Trastuzumab emtansine (T-DM1) is a conjugate of the HER2-specific monoclonal antibody trastuzumab and the cytotoxic molecule emtansine. The efficacy and safety of adjuvant therapy were demonstrated in the pivotal study KATHERINE. No significant incidence of hematological toxicities was found and no FN episodes were observed in the study group (43). T In the EMILIA study with patients with MBC, the frequency of G3/G4 neutropenia was only 2% (44). It can be concluded that hematological toxicity (neutropenia) is not a significant complication of T-DM1 therapy. There were no indications for prophylactic use of G-CSF in clinical practice.

### Trastuzumab deruxtecan

Trastuzumab deruxtecan (T-DXd) is antibody–drug conjugate (ADC), consisting of HER2-specific monoclonal antibody and topoisomerase I inhibitor deruxtecan., with the efficacy in patients with advanced or metastatic HER2-positive or HER2-low BC confirmed in the DESTINY studies program (45–47).

In T-DXd studies, neutropenia (mainly G1/G2) was a common hematologic adverse event. In the Destiny-Breast03 study, grade 3 or higher neutropenia was observed in 19.1% of patients (46). In in patients with advanced HER2-low BC, the rate of grade 3 or higher neutropenia was 13.7% compared with 40.7% in patients receiving investigator’s choice chemotherapy (48). The toxicity during T-DXd treatment is thought to be due to the deruxtecan molecule. Patients with advanced disease are initially at higher risk of developing this complication.

According to ESMO recommendations, T-DXd belongs to the group of drugs with the lowest FN risk (<10%), therefore, the use of G-CSF is not routinely recommended (2). T-DXd treatment can be continued in patients with neutrophil count >1,000 G/L. In patients with recurrent G3/G4 neutropenia, the first step is to consider dose reduction; however, the decision is made on a case-by-case basis. In patients with FN occurring during previous treatment cycles, G-CSF prophylaxis can be considered. However, it should be remembered that in the case of afebrile neutropenia, G-CSF should not be routinely used (48).

### Tucatinib

Tucatinib is a potent, selective, and reversible HER2 tyrosine kinase inhibitor, approved in combination with trastuzumab and capecitabine for the treatment of unresectable, locally advanced, or metastatic HER2-positive BC based on phase III HER2CLIMB study. Neither significant hematological toxicities (neutropenia) nor FN episodes were observed in this study (49). Therefore, tucatinib therapy does not require prophylactic G-CSF use in routine clinical practice.

### Lapatinib

Lapatinib in combination with capecitabine was registered as a second-line treatment after failure of therapy containing anthracyclines, taxanes, and trastuzumab in patients with advanced, HER2-positive BC based on the pivotal NCT00078572 study. Currently lapatinib is used less often (50, 51). In the pivotal study, the toxicity profile of lapatinib partially overlapped with AEs of capecitabine. The most common AEs included gastrointestinal disorders, rashes, and stomatitis. Neither neutropenia nor FN episodes were observed. Therefore, this therapy does not require the use of G-CSF in clinical practice.

## Hormone receptor-positive breast cancer

### CDK4/6 inhibitors

The first ciclib registered in clinical practice was palbociclib. The PALOMA pivotal study showed, that it is safe and well-tolerated therapeutic option in advanced luminal HER2-negative BC. The most common AE during palbociclib therapy is hematological toxicity. In the pivotal study, G3/G4 neutropenia was observed in 62% of patients receiving palbociclib in combination with fulvestrant and in 66,5% of patients receiving palbociclib in combination with letrozole. However, the mechanism of CDK4/6 inhibitor toxicity is different from that caused by cytotoxic drugs, and leads to inhibition of granulocyte maturation at the stage of their precursors. The highest risk of neutropenia is associated with the first 15 days of therapy, lasts for 2 treatment cycles, and decreases over time (52, 53).

Ribociclib is highly selective CDK4/6 inhibitor approved for treatment of advanced, luminal BC based on the results of the MONALEESA studies. Its acceptable safety profile was confirmed in the MONALEESA-2 study. The most common grade 3/4 adverse event was neutropenia (59.3% of patients), with median time to the onset of 16 days. Only 1.5% of patients receiving ribociclib experienced FN (54). Therefore, there is no indication for G-CSF prophylaxis in patients treated with this drug.

Another currently available CDK4/6 inhibitor is abemaciclib, which efficacy and safety were assessed in the MONARCH studies. Abemaciclib is more selective for kinase 4 than for kinase 6, which translates into a slightly different toxicity profile. The incidence of neutropenia is lower than ribociclib and palbociclib. In the pivotal study, the incidence of G3 and G4 neutropenia was 26.8% and 2,9%, respectively: FN was reported in 0.9% of patients (55).

The management algorithm for hematological toxicity, such as neutropenia, is identical for whole CDK4/6 inhibitors class. In the case of recurrent grade 3 or single episode of grade 4 neutropenia, a dose reduction is recommended. There are no indications for prophylactic use of G-CSF (56–58). Importantly, CDK4/6 inhibitors can be safely used when the neutrophil count is ≥1000 G/L.

### Alpelisib

Alpelisib is an oral selective inhibitor of the α‐isoform of class I phosphatidylinositol 3-kinase (PI3K) (59).

The efficacy of alpelisib in the treatment of advanced luminal HER2-negative BC was assessed in the SOLAR-1 study. Typical toxicities of alpelisib include hyperglycemia, rash, and diarrhea. No significant hematological toxicities were observed during the pivotal study. Therefore, therapy with this drug does not require G-CSF use (60).

## Conclusions

G-CSF is currently an integral part of perioperative chemotherapy regimens used in the radical treatment of EBC, possessing high risk of neutropenia and FN. Primary and secondary prophylaxis allows maintaining the appropriate therapy rhythm and dose intensity, which has a direct impact on outcomes. The benefits of using G-CSF has been additionally strengthened by the COVID-19 pandemic. Thanks to these observations, it is easier to make decisions about implementing FN prophylaxis, therefore reducing the risk of significant adverse effects of systemic therapy. Such procedures have translated into a significantly lower risk of FN and related complications. In radical treatment, G-CSF is used primarily in patients receiving dose-dense chemotherapy regimens and TCH+/P regimen.

Hematological toxicities related to MBC treatment have not been a significant clinical problem so far, with the exception of PTH regimen used in first-line treatment of patients with HER2-positive BC, with often prophylactic use of G-CSF. The approval of numerous new targeted therapies, including ADCs, poses new challenges for clinicians. Management algorithms of toxicities associated with the aforementioned therapies also include G-CSF, especially use as a secondary prophylaxis. Better understanding of drugs’ mechanisms of action and their characteristic side effects facilitates the implementation and continuation of treatment with modern therapies without unnecessary side effects. Preventing complications protects against premature termination of therapy, which may be a chance for a longer life for BC patients, also with advanced disease.
